# A long way to go: caspase inhibitors in clinical use

**DOI:** 10.1038/s41419-021-04240-3

**Published:** 2021-10-15

**Authors:** Shanel Dhani, Yun Zhao, Boris Zhivotovsky

**Affiliations:** 1 Institute of Environmental Medicine, Karolinska Institutet, Box 210, 17177 Stockholm, Sweden; 2 Faculty of Medicine, MV Lomonosov Moscow State University, 119991 Moscow, Russia

**Keywords:** Cell death, Immunological disorders

## Abstract

Caspases are an evolutionary conserved family of cysteine-dependent proteases that are involved in many vital cellular processes including apoptosis, proliferation, differentiation and inflammatory response. Dysregulation of caspase-mediated apoptosis and inflammation has been linked to the pathogenesis of various diseases such as inflammatory diseases, neurological disorders, metabolic diseases, and cancer. Multiple caspase inhibitors have been designed and synthesized as a potential therapeutic tool for the treatment of cell death-related pathologies. However, only a few have progressed to clinical trials because of the consistent challenges faced amongst the different types of caspase inhibitors used for the treatment of the various pathologies, namely an inadequate efficacy, poor target specificity, or adverse side effects. Importantly, a large proportion of this failure lies in the lack of understanding various caspase functions. To overcome the current challenges, further studies on understanding caspase function in a disease model is a fundamental requirement to effectively develop their inhibitors as a treatment for the different pathologies. Therefore, the present review focuses on the descriptive properties and characteristics of caspase inhibitors known to date, and their therapeutic application in animal and clinical studies. In addition, a brief discussion on the achievements, and current challenges faced, are presented in support to providing more perspectives for further development of successful therapeutic caspase inhibitors for various diseases.

## Facts

- Caspases, an evolutionary conserved family of cysteine-dependent proteases, play essential roles in modulating different biological processes including apoptosis, proliferation and inflammation.
- A plethora of diseases, such as inflammatory, neurological and metabolic diseases, and cancer, are associated with the poor regulation of caspase-mediated cell death and inflammation.
- Numerous natural and artificial caspase inhibitors have been identified and developed with the intention for therapeutical use.
- Due to the poor efficacy or toxic effects, only a limited number of synthetic caspase inhibitors have advanced into clinical trials, with none of them being successful for clinical use yet.

## Open questions

- What are the non-apoptotic and non-inflammatory roles of caspases and how do they contribute to cell function and disease? How does caspase inhibition affect these roles?
- What alternative signaling pathways are activated upon targeted caspase inhibition?
- In light of the emerging non-apoptotic and non-inflammatory roles of caspases, and the activation of alternative pathways, how can we optimally target caspases for clinical treatment without impacting one or the other?

## Introduction

**Fig. 1:**
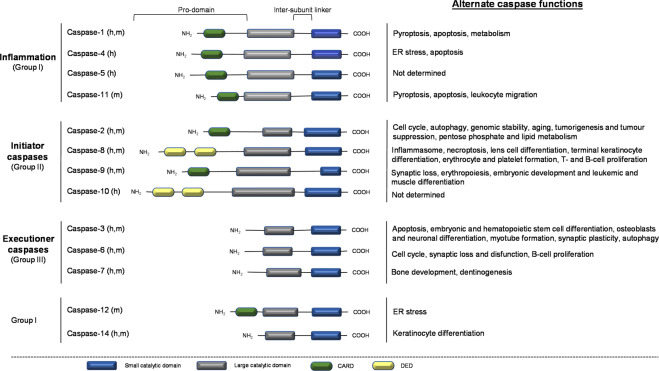
Structural and functional characteristics of caspases. The members of the caspase family are grouped based on their functional contribution in inflammation (caspases-1, -4, -5, and -11) and involvement in cell death (initiation: caspases-2, -8, -9, and -10; execution: caspases-3, -6, and -7) or by their consensus recognition sequences. Inflammatory caspases recognize the sequence Trp-Glu-His-Asp and are classified as group I. Whilst group II and group III recognize Asp-Glu-X-Asp and (Leu/Val)-Glu-X-Asp, respectively. Although caspase-12 and -14 are classified as group I, these caspases do not share the same functional role as inflammatory group I caspases. Instead, caspase-12 is believed to be a negative regulator of caspase-1 and to promote ER stress-induced apoptosis in rodents; and caspase-14 in epidermal formation. Structurally, inflammatory and initiator apoptotic caspases have a long pro-domain with a CARD or DED domain component which facilitates its recognition by a multiprotein complex or adapter molecule on death receptors, respectively, for its activation. Unlike initiator apoptotic caspases, executioner caspases lack a CARD or DED domain and require proteolytic cleavage of the inter-subunit linker for its dimerization and activation. Apart from the conventional functions of caspases, alternate roles including differentiation, migration and development in various cell types have added to their functions and have revealed the possible crosstalk between caspases and other molecular pathways. However, these roles are not yet well defined. X: represents an amino acid.

Apoptosis is a tightly regulated form of programmed cell death (PCD) that controls the activity and expression of genes and proteins involved in proliferation, differentiation, and pro-inflammatory cytokine activation [1–3]. Caspases are an evolutionary conserved family of cysteine-dependent proteases that are actively involved in the execution of apoptosis [4]. The zymogens are synthesized as catalytically inactive procaspases, composed of a large and small catalytic subunit and are activated by trans-, recruitment-, and auto-activation [4, 5]. To date, fourteen mammalian caspases have been identified and are commonly classified based on their function in apoptosis and inflammation or are grouped by their cleavage recognition sequence for substrates (Fig. 1) [6]. Structurally, inflammatory caspases contain a caspase activation and recruitment domain (CARD) apart of the long pro-domain (group I) while apoptotic initiator caspases has either a CARD or a death effector domain (DED) present in the long pro-domain (group II) (Fig. 1) [5, 7]. In contrast to initiator caspases, executioner caspases possess a short pro-domain in the absence of CARD- or DED domains (group III) (Fig. 1) [5, 7]. Despite their structural similarity, caspases have a high degree of specificity for their substrates, with a specific cleavage at aspartic acid residues.

The historic belief of caspases as mediators of apoptosis and inflammation has rendered them as attractive targets for the treatment of several diseases including neurodegeneration, inflammation, metabolic disease, and cancer [5, 8]. While the use of caspase inhibitors has undoubtedly contributed to understanding the mechanisms of cell death and inflammation in the pathophysiology of diseases, many have failed to move past clinical trials due to inadequate efficacy or an adverse safety profile because of the poor target specificity and/or non-target caspase selectivity [6, 9–12]. Despite the role of caspases in cell death and inflammation being firmly established, emerging evidence has shown the activation of alternative caspase-independent cell death processes upon caspase inhibition [13]. Likewise, an increasing number of studies have revealed that caspases and their targeted proteins mediate multiple cellular processes far beyond their apoptotic and inflammatory function, although these are not yet fully understood [13]. Evidently, caspases are multifaceted enzymes and inhibiting their activity to target their apoptotic or inflammatory functions may not be as simple.

Here, we will review the different types of caspase inhibitors, their mechanisms of action, and target specificity in several pathological disorders. Also, we will highlight emerging studies on the non-inflammatory and non-apoptotic functions of caspases, and the crosstalk between caspase signaling and other molecular pathways, in aid to provide better strategies in the development of therapeutic caspase inhibitors with enhanced long-term efficacy and reduced toxicity.

## Natural and synthetic caspase inhibitors

Since the discovery of cytokine response modifier A (CrmA) as the first caspase inhibitor, several natural caspase inhibitors were identified and numerous artificial caspase inhibitors have been developed for the pharmacological treatment of various diseases.

### Natural caspase inhibitors: viral and cellular inhibitors

- **CrmA**
  Various viruses naturally carry genes encoding caspase inhibitors to protect themselves from the hosts immune response [14–16]. CrmA was the first caspase inhibitor discovered as a product of the cowpox virus that inhibits caspase-1, also known as interleukin 1β (IL-1β) converting enzyme (ICE) [17]. CrmA belongs to the serine protease inhibitor (serpin) family and inhibits cytotoxic T cell serine protease, granzyme B, and multiple cysteine proteases [15, 17, 18]. CrmA efficiently inhibits caspases-1, -8, -10, and reduces inflammation by preventing apoptosis and the production of IL-1β and interferon γ [19, 20].
- **p35 family**
  p35, a baculovirus protein, is another viral caspase inhibitor that suppresses apoptosis in insect cells in response to viral infection [21, 22]. p35 can inhibit CED-3, one of the major protein components of the PCD pathway in *C. elegans* [21] and can bind and inhibit multiple mammalian caspases (except for caspase-9) to prevent the apoptotic response in infected cells [23]. Moreover, baculovirus p49, a homolog of p35, was discovered as a substrate inhibitor which prevents apoptosis in vivo by inhibiting p35-insensitive initiator caspase [24].
- **Inhibitor of apoptosis (IAP) proteins**

IAP genes were first identified from the *Cydia pomonella* granulosis virus for being able to rescue infected cells from apoptosis [25]. Subsequent studies identified a group of IAP family members as a cellular component expressed in yeast, *C. elegans* and mammals [26]. To date, eight IAP family members, NAIP, XIAP, cIAP1, cIAP2, survivin, BRUCE, livin (ML-IAP, KIAP), and ILP-2, have been identified in humans [27]. Of all the IAPs, XIAP is the most studied because of the direct binding and inhibition of caspase−3, −7, −9 [28–31]. Similarly, cIAP1 and cIAP2 are two caspase inhibitors which bind and inhibit caspase−3/−7 directly [32].

### Synthetic caspase inhibitors

The dysregulation of apoptosis and inflammation plays an important role in the development of numerous diseases. A plethora of artificial caspase inhibitors has been developed as a potential means of treatment for cell death-related diseases. In general, synthetic caspase inhibitors are classified as peptide-based and non-peptide compounds (Table 1).

- **Peptide-based inhibitors**

**Table 1:** Commonly studied synthetic caspase inhibitors.

Types	Caspase inhibitors	Caspases targeted	References
Peptide-based inhibitors	Ac‐IETD‐CHO	Caspase-8	[55]
	Ac‐YVAD‐CHO	Caspase-1	[55]
	Ac‐DEVD‐CMK	Caspase-3	[152]
			[153]
	Z-VAD-FMK	Broad spectrum, e.g., caspase-2, -3, -8 & -9	[154]
			[63]
	Z-YVAD-FMK	Caspase-1, -4	[155]
	Boc-D-FMK	Broad spectrum, e.g., caspase-3, -7–9, poor inhibitor of caspase-2, -5, -6, -10	[154]
			[156]
	TRP-601	Caspase-2	[157]
	Q-VD-OPh	Broad spectrum, e.g., caspase-1, -2, -3, -6, -8. -9	[154]
Peptidomimetic inhibitors	VX-765 (belnacasan)	Caspase-1	[12]
	VRT-043198 (the active metabolite of VX-765)	Caspase-1, -4	[158]
	VX-740 (pralnacasan)	Caspase-1	[39]
	IDN-6556 (emricasan, PF-034911390)	Broad spectrum, e.g., caspase-3, -7, -8	[159]
			[160]
	VX-166	Broad spectrum, e.g., caspase-1, -3, -7	[161]
	M826	Caspase-3	[162]
	M867	Caspase-3	[162]
Non-peptidic compounds	QPI-1007 (cosdosiran)	Caspase-2	[163]
	NCX-1000	Caspase-3, -8, -9	[12]
			[164]
	Isatin sulfonamides	Caspase-3, -7	[165]
Allosteric caspase inhibitors	FICA	Caspase-3, -7	[166]
			[44]
	DICA	Caspase-3, -7	[166]
			[44]

The first synthetic caspase inhibitors were developed as peptides, on which the aspartic acid was modified with a reactive electrophilic group, enabling the inhibitors to covalently link with the nucleophilic active thiol site of the enzyme in a reversible/irreversible way [8, 11].

Peptides modified with aldehyde, ketone or nitrile groups are reversible inhibitors which bind to the catalytic cysteine and can be hydrolyzed without altering the enzyme structurally [15, 33]. The peptide moiety is the determinant for their selectivity to specific caspases [34]. Ac-YVAD-CHO is a potent inhibitor of caspase-1 containing a pro-IL-1β cleavage site, while Ac-DEVD-CHO has a stronger selectivity for caspase-3 due to its PARP cleavage site [34]. However, the therapeutic utility of aldehyde-based caspase inhibitors is limited by poor membrane permeability, stability, and potency. Alternatively, peptides linked to α-substituted ketone groups, e.g., chloro-(CMK) and fluoro-(FMK) methyl ketones, acylomethyl ketones, and phosphinyloxy methyl ketones, enable the formation of the thiomethyl ketone with the catalytic site of cysteine to irreversibly inactivate caspases [15]. Amongst ketone-modified peptides, FMK linked inhibitors, e.g., Z-VAD-FMK, with improved permeability, failed as a potential therapeutic agent due to high toxicity in vivo [35]. Q-VD-OPh, a broad-spectrum caspase inhibitor, was synthesized with enhanced efficacy, permeability, and nontoxic effects in vitro even at high (500 µM or 1000 µM) concentrations [36] and effectively maintains T cell ratios while decreasing viral loads in SIV-infected rhesus macaques [37]. In comparison with other carboxyterminal-conjugated caspase inhibitors, Q-VD-OPh provides valuable insight regarding the therapeutic use of artificial caspase inhibitors in vivo.

- **Peptidomimetic inhibitors**
  Peptidomimetic inhibitors have been developed due to the pharmacological drawbacks of peptidic inhibitors such as poor stability, low potency and rapid metabolism [8, 38]. As an irreversible pan-caspase inhibitor, IDN-6556 (emricasan) was developed for the treatment of liver diseases. The preclinical and clinical studies have shown its efficacy, however, some side effects triggered by extended treatment with IDN-6556 were exposed and its clinical development was terminated due to undisclosed reasons [12]. VX-740 (pralnacasan), a peptidomimetic inhibitor for caspase-1, was tested in clinical trials [39]. Orally used VX-740 possessed significant potency for rheumatoid arthritis (RA) and osteoarthritis (OA) treatment [40, 41]. However, the clinical trials for VX-740 were terminated due to the liver toxicity induced by high doses in animal models [12]. VX-765 (belnacasan), a reversible caspase-1 inhibitor, is more potent than pralnacasan for the treatment of inflammatory diseases [12]. Nevertheless, clinical trials for VX-765 were terminated because of its liver toxicity. Despite the promise shown by these caspase inhibitors, very few have entered clinical trials because of the gap between treatment regimens in animal and clinical studies.
- **Non-peptidic compounds**
  Limitations of peptidomimetic inhibitors have facilitated the synthesis of other small-molecule caspase inhibitors. Isatin derivatives (e.g., sulfonamides) are a representative group of potent caspase-3/−7 inhibition and are of great interest for drug development [42]. Some nitric oxide donors, e.g., NCX-1000, have shown better potency against liver disease in clinical trials [12, 43]. However, the adverse effects such as reduced portal pressure resulted in its discontinuation, indicating a long way to go before fully applying non-peptidic caspase inhibitors for clinical use.
- **Allosteric caspase inhibitors**

Unlike most of the peptidomimetic or small-molecule inhibitors which target the active site of caspases, allosteric caspase inhibitors target the allosteric site of caspases. Two groups of compounds, 5-fluoro-1H-indole-2-carboxylic acid (2-mercaptoethyl) amide) and 2-(2,4-dichlorophenoxy-N-(2-mercapto-ethyl)-acetamide), were identified as allosteric caspase inhibitors which target caspase-3/-7 [44]. The chemical structure of allosteric inhibitors allows it to advantageously bind to the dimer interface of caspases and inhibit them. No allosteric caspase inhibitor has progressed into clinical use, indicating that more optimizations are needed [12].

## Caspase inhibitors for diseases: animal models and clinical trials

A number of disorders such as neurodegenerative disorders [45], liver diseases [46], and cancer [2] are strongly associated with abnormal caspase activity and apoptosis (Table 2). Given the considerable challenges still faced with the development of caspase inhibitors, no synthetic drugs have yet been applied for the treatment of such diseases [8]. Nevertheless, it is worthwhile to probe into the studies on caspase inhibitors, which will provide more insights into the research and development of therapeutic agents for various diseases.

**Table 2:** Caspase-associated diseases and caspase inhibitors used in animal models and clinical trials.

Disease categories	Diseases/disorders		Caspase inhibitors	Animal models	Clinical trials	References
Inflammatory diseases	Psoriasis		VX-765		Completed Phase II trial	[49]
			Ac-YVAD-CMK	Imiquimod-induced mice	N/A	[51]
	RA and OA		VX-740	Collagen-induced mice SCW-induced mice	Phase II trial	[40, 41]
			Z-VAD-FMK	Rabbit with ACLT transection	N/A	[55]
	Sepsis/septic shock (endotoxic shock)		Z-VAD-FMK, M920, M791, Z-LEHD-FMK, VX-166	CLP-induced mice/rats, LPS-induced mice/rats	N/A	[58, 60, 62, 161]^,^
Neurological diseases	Traumatic central nervous system (CNS) injuries		Z-DEVD-FMK, Ac-DMQD-CHO, Z-LEHD-FMK, Q-VD-OPh, Boc-D-FMK, L-826791	TBI-induced rodents, SCI-induced rodents	N/A	[68–73]
	Epilepsy		VX-740	kainic acid-induced seizure		[78]
			VX-765		Phase IIa trial	[79]
	Neurodegenerative diseases	AD	Q-VD-OPh, Z-DEVD-FMK, VX-765	TgCRND8 mice, Tg2576 mice, J20 mice	N/A	[84, 86, 87]
		ALS	Z-VAD-FMK, XIAP, p35	SOD1-mutated mice		[91, 92]
		HD	Q-VD-OPh, M826	3NP/malonate- injected mice		[95, 96]
		PD	Q-VD-OPh	MPTP-induced mice		[95]
Metabolic diseases	Obesity		VX-740, Ac-YVAD-CMK	ob/ob mice, High fat diet-fed obese LDLR ^-/-^ mice	N/A	[104, 107]
			Ac-DEVD-CHO	N/A (ex vivo)		[102]
	Diabetes		Z-VAD-FMK, EP1013, IDN-6556	Islet-transplanted mice	N/A	[118, 119]
	Liver disease		IDN-6556, VX-166, GS-9540		Phase II trial	[123, 125, 129]
Cancer	Breast cancer, lung cancer, etc		Z-VAD-FMK, M867, NH-23-C2	N/A (in vitro studies)		[136–138]
Additional diseases	AIDS		Q-VD-OPh	SIV-infected rhesus macaques		[37]

### Inflammatory diseases

Since caspases play an important role in modulating inflammation and inflammatory disorders, caspase inhibitors have been widely utilized to study inflammatory diseases in animal models and clinical trials.

- **Psoriasis**
  Psoriasis is a chronic inflammatory skin disease, whose pathogenesis is still poorly understood [47, 48]. The increased levels of caspase-1 and multiple proinflammatory cytokines such as IL-1β and TNF-α have been linked to the development of psoriasis, suggesting that caspase-1 might be a crucial target for the treatment [49].
  VX-765, a caspase-1 inhibitor, was used to treat psoriasis in Phase I and II clinical trials (clinical trial: NCT00205465) [6, 49, 50], which was completed in 2005. However, the subsequent results have yet to be disclosed. Another caspase-1 inhibitor Ac-YVAD-CMK was shown to attenuate the imiquimod-induced psoriasis-like phenotype in a mouse model [51]. Despite the positive inhibition of psoriasis with Ac-YVAD-CMK more animal and clinical evidence is required for its potential use.
- **RA and OA**
  Arthritis is an inflammatory disorder that affects the joints, with RA and OA being the most common types worldwide [52]. Caspase inhibitors have been investigated for potential treatment for arthritis. In a collagen-induced arthritis mice model, pralnacasan, a reversible caspase-1 inhibitor, significantly delayed the onset of inflammation and attenuated the disease by 50–70% at doses of 50–100 mg/kg b.i.d (twice of a day) [53]. The blockade of caspase-1 was shown to reduce joint swelling and severe cartilage damage in streptococcal cell wall (SCW)-induced chronic arthritis mice. In collagenase-induced female mice and a STR/1N male mouse strain, which spontaneously develops OA, pralnacasan reduced joint damage [41]. In Phase I clinical trials, pralnacasan showed a 50% oral bioavailability [54]. In a Phase II trial with 285 RA patients, pralnacasan significantly inhibited the inflammatory response without notable adverse side effects [40]. However, these clinical trials were shut down due to liver toxicity shown in long-term animal studies [12]. In a rabbit model of anterior cruciate ligament transection (ACLT)-induced OA, Z-VAD-FMK reduced the level of active caspase-3 in chondrocytes and inhibited the cleavage of PARP, resulting in the alleviation of cartilage lesions and confirmed the role of cell death in OA pathogenesis [55].
  A major concern of advancing peptidomimetic caspase inhibitors for clinical trials is the considerable organ toxicity in animal models. The pharmacokinetic liabilities of these inhibitors have limited their efficacy in vivo and multiple factors including the poor membrane permeability, metabolic stability, and toxicity have restricted the therapeutic effect [12]. Further optimization is needed regarding the structure of these compounds as well as the regimen of treatment.
- **Sepsis/septic shock (endotoxic** **shock)**

Sepsis or septic shock (endotoxic shock) is an inflammatory response of the host against severe infection which results in serious cell and organ injury [56, 57]. Sepsis has been associated with excessive lymphocyte apoptosis in the thymus and spleen with notable lymphopenia [58]. Therefore, inhibition of caspases may function as a potential therapy via blocking sepsis-induced lymphocyte apoptosis [59]. The effectiveness of broad-spectrum caspase inhibitors Z-VAD-FMK and M920, and selective caspase-3 inhibitor M791 in blocking lymphocyte apoptosis and improving survival of sepsis was shown using a mouse cecal ligation and puncture model [60, 61]. Treatment with Z-VAD-FMK facilitated the accumulation of myeloid-derived suppressor cells and alleviated lipopolysaccharide-induced endotoxic shock in mice [62]. Local injection of the caspase-9 inhibitor Z-LEHD-FMK in a CLP-induced septic murine model decreased apoptosis in resident tissue cells and improved the survival of mice [58]. The administration of VX-166, a broad-spectrum caspase inhibitor, significantly improved survival in animal models with sepsis with less adverse immunosuppressive effects suggesting that this compound might be a strong candidate for the treatment of sepsis in humans [63]. Despite the promise, without sufficient animal studies, subsequent clinical studies cannot investigate the therapeutic use for sepsis treatment.

### Neurological diseases

The involvement of caspases in various neurological diseases have indicated the potential role of caspases as an encouraging therapeutic target.

- **Traumatic central nervous system (CNS) injuries**
  Traumatic injuries to the CNS, including traumatic brain injury (TBI) and spinal cord injury (SCI), is initiated with primary damage which results in a cascade of events that lead to sustained impairments and disabilities, and prolonged neurological degeneration [64, 65]. Apoptosis plays a critical role in contributing to the cell loss following CNS trauma, and its inhibition with caspase inhibitors has been emerging as a prospective treatment of CNS injuries [66].
  Numerous studies have demonstrated the neuroprotective effect of the caspase-3 inhibitor Z-DEVD-FMK on rodent models with TBI (intracerebral infusion, 240 ng or 480 ng, 1 µl/h) [67] and SCI (i.p, 800 or 1600 µg/kg) [68]. Treatment with the caspase-3 inhibitor Ac-DMQD-CHO (i.v, 1 mg/kg) significantly attenuated apoptosis and improved the functional outcome in a rat model with SCI [69]. The application of caspase-9 inhibitor Z-LEHD-FMK (i.v, 0.8 µM/kg) significantly blocked apoptosis and reduced the expansion of lesion in a SCI rat model [70]. The administration of broad caspase inhibitors, Q-VD-OPh (i.p, 0.4 mg/kg) [71] and Boc-D-FMK (intracerebroventricular injection, 300 nmol in 5 µl, 0.5 µl/h) [72], also demonstrated favorable effects on neuroprotection in animal models with traumatic CNS injuries.
  Additionally, a caspase-3 inhibitor from Merck Frost Canada, L-826791, was revealed to reduce apoptosis in the hippocampus and piriform cortex in preclinical trials for the treatment of brain injury [73]. However, no follow-up clinical studies were conducted. Due to differences in the approach, period, and dosage of drug administration, it is difficult to conduct a direct intercomparison of the efficacy among the different caspase inhibitors. Nevertheless, the neuroprotection of peptide-based caspase inhibitors on CNS trauma in animal models indicates the potentiality of developing caspase inhibitors as a therapeutic agent for this disease.
- **Epilepsy**
  Epilepsy is a chronic neurological disorder with a tendency to develop into epileptic seizures [74]. Epilepsy is often associated with other neurological comorbidities, however, the pathophysiology underlying the onset and development of epilepsy is not fully understood [74, 75].
  The elevated level of IL-1β in brain areas has been identified in both rodents and humans with epilepsy, and the prominent anticonvulsant effects induced by the inhibition of IL-1β suggests that this cytokine might be a potential target for the treatment of epilepsy [76, 77]. The administration of caspase-1 inhibitors, VX-740 or VX-765, suppressed seizure-induced IL-1β production in the hippocampus of rat models and led to a delay in seizure onset and half reduction in seizure duration [78]. Moreover, a 15.6% reduction in seizure rate (7.0% in the placebo group) was shown with the treatment of VX-765 in a Phase IIa clinical trial and a delayed beneficial effect of this compound was indicated by the animal model and the post hoc analyses after discontinuing the administration [79]. In terms of safety and tolerability, 72.9% subjects in the VX-765 group showed at least one treatment-emergent adverse event (TEAE) during therapy compared with 83.3% subjects in the placebo group. However, the rate of serious TEAE occurrence was 6.3% in VX-765-treated patients compared with 0% of placebo subjects [79]. Thus, further studies are required to confirm these observations and to demonstrate the comprehensive safety assessments before fully developing it for clinical use.
- **Neurodegenerative diseases**
  The prevalence of neurodegenerative diseases, including Alzheimer’s disease (AD), amyotrophic lateral sclerosis (ALS), Huntington’s disease (HD), Parkinson’s disease (PD) and few others, is increasing rapidly and results in a significant proportion of morbidity and mortality worldwide [80]. However, few or no effective cure options for neurodegenerative disorders have been fully developed [81, 82]. All neurodegenerative diseases are characterized by the progressive loss of a particular subset of neurons associated with neuronal death, one of which is apoptosis [80, 83]. As the core mediators of apoptosis, caspases have been emerging as potential therapeutic targets for the treatment of neurodegeneration with several caspase inhibitors investigated in neurodegenerative animal models.
- **Alzheimer’s disease**
  The activation of caspases including caspase−3, −6, −8, −9, and cleavage of crucial proteins, such as amyloid precursor protein and tau, have been implicated in human brains with AD [84, 85]. A pilot study using a suitable animal model of AD that displays caspase activation and cleavage of AD-related proteins demonstrated that chronic treatment with Q-VD-OPh prevented the activation of caspases, cleavage of tau protein and limited the tau-associated pathological changes [84]. Additionally, inhibition of caspase-3 using Z-DEVD-FMK was proved to rescue the Alzheimer-like phenotypes in mice models, uncovering the vital role of caspase-3 in driving synaptic failure and contributing to the AD-associated cognitive dysfunction [86]. VX-765 has also been shown to alleviate cognitive impairment and delay cognitive decline in mouse models of AD, suggesting that VX-765 may be an effective drug for the prevention of onset of AD-associated cognitive deficits [87, 88].
- **Amyotrophic lateral sclerosis**
  As one of the most frequent neurodegenerative diseases, ALS is an idiopathic and fatal disorder that affects the human motor system [89]. The missense mutations on the gene encoding Cu/Zn superoxide dismutase 1 (SOD1) were discovered in subsets of familial cases with ALS, providing a promising direction to elucidate the underlining mechanisms and, the development of targeted treatments [90]. Using a transgenic mouse model of ALS produced by the mutation in *SOD1* gene, the administration of Z-VAD-FMK inhibited caspase-1 activity and delayed the onset and mortality of ALS [91]. To elucidate the function of caspase-9 in ALS, XIAP (a mammalian inhibitor targeting caspase−3, −7, −9) and p35 (a baculoviral caspase inhibitor not targeting caspase-9) were administrated in transgenic ALS mouse models, and an attenuation effect on the disease progression was shown only in XIAP-treated mice while the delayed onset effect was only induced by p35, suggesting a critical role of caspase-9 in the development of ALS [92].
- **Huntington’s disease**
  HD is characterized by a CAG repeat expansion in the huntingtin (*HTT*) gene on chromosome 4 and is the most common autosomal-dominant progressive neurodegenerative disorder in developed countries without an effective treatment so far [93, 94]. The establishment of HD animal models by injection of mitochondrial toxins, 3-nitropropionic acid (3NP) and malonate, provides a useful tool to investigate the pathological mechanisms. Two rat models treated with 3NP suggested a significant elevation of the active form of caspase-8, which induced truncation of Bid, a proapoptotic protein in the Bcl-2 family, leading to its translocation from the cytoplasm to the mitochondria and activation of mitochondria-mediated cell death [95]. The administration of Q-VD-OPh was shown to effectively inhibit caspase activation and largely reduce striatal lesions produced in rat models of HD [95]. The treatment with M826, a caspase-3 inhibitor, was able to suppress caspase-3 activation and provide neuroprotective effects in a rat model with HD [96].
- **Parkinson’s disease**

PD is a progressive neurological disorder that is characterized by the degeneration of dopaminergic (DA) neurons in the mesencephalon with prominent neuronal loss [97, 98]. Despite the controversy of the contribution of apoptosis to neuronal loss in PD, degeneration of DA neurons by apoptosis has been suggested [99]. Additionally, 1-methyl-4-phenyl-1,2,3,6-tetrahydropyridine (MPTP), a key reagent for establishing animal models of PD, was found to activate caspase-3, −8, −9, and induce the cleavage of Bid resulting in increased mitochondrial-mediated dopaminergic cell death [100]. Notably, the above changes were inhibited in transgenic mice expressing baculoviral caspase inhibitor p35 in the neurons [100]. The application of Q-VD-OPh in MPTP-treated mice induced a significant reduction of dopamine depletion in the striatum and inhibited the loss of dopaminergic neurons in the substantia nigra [95].

Overall, the studies of applying caspase inhibitors for the treatment of neurodegenerative diseases, so far, have only focused on the animal models, without substantial evidence from clinical trials yet. Despite the increasing amount of animal studies demonstrating promising results of several caspase inhibitors, the current developmental process of the caspase inhibitors as a therapeutic agent for neurodegenerative diseases is still in the early phase with many unsolved biological issues. The utility of different animal models in therapeutic studies differs making it difficult to determine a best model (if any) to represent the clinical situation; and the biological studies of the involvement of caspases/apoptosis in chronic neurodegenerative disease are not as established as that in acute neuronal injuries [73]. Therefore, it is critical to address the above-mentioned questions to promote animal studies into feasible clinical trials and further into the development of therapeutic caspase inhibitors for neurological diseases.

### Metabolic diseases

An extensive body of evidence have implicated metabolic inflammation and cell death as key players in the pathogenesis of multiple metabolic diseases such as obesity, diabetes, and liver disease.

- **Obesity**
  Obesity occurs from increases in adipocyte size (hypertrophy) and number (hyperplasia) that results in local inflammation and the accumulation of lipids in non-adipose tissues such as the liver and skeletal muscle [101, 102]. Adipocyte hypertrophy occurs to a certain extent before it induces different modes of PCD (necroptosis, pyroptosis and apoptosis) to maintain or increase its lipid-storage capacity, which promotes the infiltration of adipocyte tissue macrophages that are tasked to remove the apoptotic bodies and lipid-droplet remnants, further promoting adipose tissue inflammation [102]. The enhanced secretion of pro-inflammatory cytokines during adipocyte hypertrophy occurs via the activation of the caspase-1/inflammasome and contributed to insulin resistance in several mice models (*IL-1β*^*-/-*^, *Caspase-1*^*-/-*^, *NLRP3*^*-/-*^, db/db, and ob/ob mice) [103, 104]. Notably, ob/ob mice treated with pralnacasan (100 µM) for 2 weeks showed a significant improvement of insulin sensitivity and a slight reduction in adipose mass and total body weight [104].
  Other caspases have also been shown to be associated with obesity-induced conditions [105, 106]. A role for caspase-2 in adipose tissue proliferation and lipoapoptosis susceptibility was assessed on differentiated 3T3-L1 cells and showed significant reductions in caspase-3/-7 activity in the presence of Z-VAD-FMK following palmitate treatment [105]. Likewise, Ac-DEVD-CHO demonstrated an increased mRNA expression of insulin signaling components (GLUT4, IRS1, IRS2) in human adipose tissue treated with anti-inflammatory cytokines TNF-ɑ or IL-6 [102]. However, these studies have utilized caspase inhibitors as a proof of concept to further understand the relationship between caspases and disease progression in experimental models and were not intended as treatments for the disease. Additionally, despite the link between caspases and metabolic inflammation, the defined role of these caspases remains unclear.
  In the context of limited adipocyte hypertrophy, the relationship between adipose tissue inflammation and turnover by targeting TGF-β–activated kinase 1 (TAK1) was investigated and showed significant reductions of caspase-3 activity in the presence of Z-VAD-FMK in TAK1-deficient adipocytes from mice fed a high-fat diet in vitro [101]. However, the study primarily focused on the proinflammatory and cell death functions of TAK1 and not the caspase-induced cell death or inflammatory mechanisms.
  While several studies performed on knockout/null mice models fed a high-fat diet have shown the therapeutic potential of caspase-1, -8, -11, -12 deficiencies in obesity, it is unclear if or how the use of therapeutic inhibitors would translate in a clinical setting. A recent study on high fat diet-fed obese LDLR^-/-^ Leiden mice (presenting obesity-associated hypertriglyceridemia, hypercholesterolemia, hyperglycemia, and hyperinsulinemia) administered with the caspase-1 inhibitor, Ac-YVAD-CMK (40 mg/kg, i.p. once daily), over a period of 12 weeks showed a delayed progression of obesity-associated liver disease and insulin resistance with improved adipose tissue inflammation, but no changes to body weight or dyslipidemia were observed [107]. While this study did therapeutically utilize the inhibitor, the authors only evaluated the physiological markers and not the underlying molecular targets of the inhibitor. Therefore, its caspase target specificity and possible activation of feedback mechanisms remains ambiguous.
- **Diabetes**
  Diabetes causes a number of physiological abnormalities in both type 1 (insulin-dependent) and type 2 (insulin-resistant) diabetic patients with diabetic retinopathy being prevalent in all patients [108]. Although glycaemic control has been shown to prevent the development of diabetic retinopathy in a number of patients, it is very difficult to accomplish and maintain in the majority of patients [108].
  The activation of several caspases (-1, -2, -3, -4, -6, -8, -9) was associated with the progression of diabetic retinopathy. An elevation of inflammatory caspases-1, -4, and -5 and executioner caspase-6 was shown in the retinas of diabetic mice over an 8-month period [109]. At the same time, the activity of initiator apoptotic caspases-8 and -9 gradually decreased through the duration of the disease. Despite this, caspase-3 activity levels increased over the time course [109]. These findings indicate that the activation of caspase-3 was independent of caspase-8 and -9, and perhaps activated by another pathway such as the p38 MAPK, which has been shown to be involved in caspase-3 processing during apoptosis in several cancer cell types [109–114].
  A preclinical study using minocycline, a tetracycline antibiotic, reported the inhibition of caspase-1 activity in the retinas of diabetic mice [115]. Whilst glucose blood levels were not altered, caspase-1 activity was inhibited more significantly by the drug when administered three times a week than when it was administered daily over a 2-month period. Subsequently, diabetic mice administered minocycline over 6 months (three times a week) were shown to inhibit degeneration of retinal capillaries and completely inhibit caspase-1/-3 activities in retinal Müller cells in vitro (high glucose), demonstrating that the antibiotic does not inhibit caspase-1 activity only [115]. While these findings are promising, further preclinical studies are required to determine if the effects of minocycline are reproducible and to understand its selectivity for caspases. Additionally, while the clinical use of minocycline is well tolerated, there are reports of adverse effects, albeit at high doses (50 mg/kg), thus the long-term effects of minocycline in diabetes need to be examined [5].
  In addition to type 1 diabetes therapy, islet transplantation has become a promising treatment for many viable patients [116, 117]. However, reductions in graft survival and function remains a significant challenge [116]. Several preclinical studies have evaluated the efficacy of various caspase inhibitors in improving islet engraftment and function. Z-VAD-FMK was shown to induce a significant improvement in islet mass function in renal subcapsular and intraportal transplantation by pretreatment with the inhibitor (2 h before transplant) and for five days thereafter (10 mg/kg.b.w, s.c) and was able to maintain graft function 1-year post-transplant [116]. Despite the beneficial effects of inhibitor therapy, its activity is non-specific for caspases. Therefore, the authors investigated a specific dipeptide caspase inhibitor EP1013 (Z-VD-FMK) using the same experimental approach [118]. Compared to Z-VAD-FMK, EP1013 (10 mg/kg.b.w, 3 mg/kg.b.w or 1 mg/kg.b.w, s.c.) was more effective in preserving transplanted islet graft and function within portal circulation, possibly due to Z-VAD-FMK having activity against cysteine proteases such as cathepsin and calpain [118]. Importantly, in the chronic study period, some of the experimental animals treated with EP1013 died from varying causes [cirrhosis (63 weeks), peritoneal infection (54 weeks), epithelial tumor (renal origin, 72 weeks), and lymphosarcoma (73 weeks)], although the exact cause of death is unknown. Based on the short-term findings, the efficacy of the inhibitor in human islets for transplantation (3 mg/kg.b.w, s.c) was evaluated [118]. While the results were comparable between the control and experimental groups, animals treated with the inhibitor displayed improved islet yields and reached euglycemia quicker than the control groups [118]. In another study, a combination therapy with EP1013 (3 mg/kg.b.w s.c., days 0–10) and an immunosuppressive agent, CTLA4-Ig (0.25 mg i.p., days 0, 2, 4, and 6 post-transplant) was assessed in immunocompetent diabetic mice with the aim of evaluating the efficacy of the inhibitor with regard to immune tolerance [119]. Despite EP1013 being an inhibitor for caspases-1, -3, -6, -7, -8, -9 [118, 119], treatment with the inhibitor alone did not prolong graft survival. Although the specificity of the inhibitor or measures of other cell death markers were not confirmed, these findings could suggest that there may be alternative cell death pathways that led to poor graft survival rates. However, treatment with CTLA4-Ig alone, or in combination with EP1013 showed improvements to graft survival, more significantly in combination therapy, and reduced functional alloreactive T-cell responses in the grafts [119].
  A more promising irreversible pan-caspase inhibitor, IDN-6556, enhanced diabetes reversal rates post-transplant in both mouse and human islet transplants into immunodeficient diabetic mice (10 mg/kg or 20 mg/kg, i.p. twice daily, once pre-transplant and 7 days post-transplant) [117]. The authors further demonstrated improved glucose tolerance and graft survival 1-month post-transplant [117]. However, similar to the investigation of Ac-YVAD-CMK in obesity, there is a lack of understanding in the specificity of IDN-6556 and its long-term effects.
- **Liver disease**

The increasing prevalence of obesity and type 2 diabetes has led to a concurrent incidence in non-alcoholic fatty liver disease (NAFLD) and is now the leading cause of chronic liver disease in western countries [120, 121]. NAFLD exists in two forms, as non-alcoholic fatty liver (NAFL) and non-alcoholic steatohepatitis (NASH) [120]. NAFL is characterized by the hepatic accumulation of lipids (steatosis) without hepatocellular injury and progresses to a more severe form of the disease, NASH, that presents with hepatocellular apoptosis, inflammation, and fibrosis, which can further progress to end-stage liver disease, cirrhosis, and hepatocellular carcinoma (HCC) [121, 122].

While there is no established pharmacological treatment for NAFLD, several experimental models have implicated extrinsic apoptosis as the predominant cause of injury and hepatocyte death and has been shown to be significantly upregulated in correlation with the severity of NASH [123]. Consequently, caspase inhibitors have been proposed as an attractive therapeutic target for NASH. However, surviving hepatocytes in chronic liver injury have shown to exhibit enhanced anti-apoptotic defense, limiting the proper removal of premalignant cells, which could potentially lead to the development of HCC [123]. Thus, the use of caspase inhibitors may provide little benefit or even exacerbate disease progression [122, 123], which can contribute to the challenge of safety and efficacy with caspase inhibitors and therefore merits consideration.

Evaluation of the pan-caspase inhibitor, emricasan, which was also proposed to be beneficial in diabetic treatment, was shown to efficiently inhibit apoptosis, liver injury, and inflammation in several preclinical models [9, 124]. Despite emricasan being a good therapeutic candidate for NASH, due to its preferential distribution to the liver, well-tolerance and potency, and its success in experimental models and in NASH patients (25 mg, twice daily, 28 days) [125], its application failed to translate in Phase II clinical trials. In a randomized, double-blind, placebo-controlled study, treatment with emricasan (5 mg/50 mg, twice daily, 72 weeks) did not improve liver histology in patients with NASH fibrosis but worsened tissue fibrosis and ballooning. In another Phase II trial in patients with NASH-related cirrhosis and severe portal hypertension, emricasan (5/25/50 mg, twice daily, 24 weeks) again showed no improvement in portal hypertension across the different dosages [126]. Notably, serum levels of alanine (ALT) and aspartate (AST) aminotransferase were directly proportional to caspase-3/-7 activity, with increasing activity over time [126, 127]. In a more recent clinical trial, emricasan proved ineffective in the treatment of decompensated NASH cirrhosis (5/25 mg, twice daily, 48 weeks) and against caspase activity (5 mg) [128]. Despite the uniform treatment regimen (dosage and frequency) with emricasan, its failure for long-term use remains unknown. Perhaps, over time the cells become tolerant to treatment with the inhibitor or may even have led to the activation of alternative cell death pathways. Nonetheless, a better understanding of its pharmacokinetics is needed to improve its therapeutic approach.

Besides emricasan, other caspase inhibitors have been assessed in the treatment of NASH. VX-166 was shown to inhibit hepatic apoptosis thereby suppressing the development of fibrosis in a preclinical model of NASH (6 mg/kg, orally once daily, 8 weeks) [123]. However, no improvements to liver injury were observed [123]. GS-9450, an irreversible inhibitor of caspase-1, -8, -9, reduced liver enzyme levels (ALT and AST) in a Phase II trial in patients with NASH (1/5/10/40 mg, once daily, 4 weeks) [129]. Notably, levels of ALT and AST were reduced further at higher dosages [129]. However, the use of GS-9450 was terminated in a Phase II clinical trial in hepatitis C patients (10/40 mg, once daily, 24 weeks, every other day at week 25, and then once every 3 days in week 26) due to drug-induced liver toxicity concerns (NCT00874796). Despite the promise shown by these inhibitors, there is not enough evidence to advocate for their use in the treatment of NASH, which may be a result of the lack of specificity of the inhibitors.

### Cancer

The hallmarks of cancer described by Hanahan and Weinberg has proved to be immensely influential in our understanding on the complexities of cancer biology and its relation to drug design [130]. These hallmarks consist of ten physiological alterations including evading cell death, namely apoptosis, by disrupting the balance between pro- and anti-apoptotic proteins, supressing caspase function and inhibiting death receptor signaling [2, 3, 130, 131]. Regardless of its complexity, advances in cancer treatment, mainly with apoptosis-based agents, have resulted in a decline in cancer mortality over the years [132]. However, despite significant efforts of several clinically tested apoptotic-based drugs, only one BH3-mimetic drug, venetoclax, which selectively inhibits the Bcl-2 protein that is overexpressed in most B-cell lymphoid malignancies, has been approved for treatment [133]. While the promise of venetoclax has been demonstrated, several key challenges remain, including its sensitivity towards a small subset of B-cell malignancy subtypes and drug resistance in a proportion of patients. Nevertheless, numerous efforts to enhance the efficacy of venetoclax in combination with chemotherapeutics, monoclonal antibodies, and kinase and protease inhibitors are currently ongoing [133, 134]. Additionally, other BH3-mimetics, which selectively target Mcl-1 and Bcl-xL against various types of malignancies, including solid tumors, are currently under investigation [134]. While there is hope for Mcl-1 inhibitors to be established as a treatment, Bcl-xL inhibitors have failed to enter clinical trials due to their toxicity.

Preclinical studies with caspase inhibitors have also demonstrated the ability to sensitize cells to anti-cancer treatments by a targeted induction of alternate cell death processes in caspase inhibited conditions. Z-VAD-FMK (20 µM) promoted the infection of monocyte chemoattractant factor-7 and MDA-MB-231 breast cancer cells with oncolytic herpes simplex virus-1 (HSV-1) and suppressed the growth of infected-tumor cells [135]. Similar results were demonstrated in MDA-MB-231 breast and H460 lung cancer cells, in which Z-VAD-FMK (50 µM) enhanced the radio-senitization of the cells and induced autophagic cell death in vitro [136]. Additionally, the inhibitor significantly slowed down tumor growth (breast and lung xenografts) and tumor vasculature in both in vitro and in vivo (2 mg/kg, i.p. once daily, 1 week) models [136]. Likewise, the caspase-3 inhibitor, M867, reduced tumor progression and vasculature in H460 lung cancer cells in vitro (5 nM and 10 nM) and in vivo (2 mg/kg, i.p. once daily, 1 week) in response to ionizing radiation [137].

Despite the abundant caspase-based inhibitors being developed for cancer treatment, very few candidates move beyond preclinical trials. Additionally, these molecules have a very poor success rate in clinical trials, again due to the lack of specificity, efficacy, and, importantly, susceptibility to drug resistance. However, in a recent study, the caspase-2 inhibitor NH-23-C2, was shown to selectively inhibit its activity and prevent MDM2 cleavage in reversine-treated HCT-116 colon cancer cells and demonstrated no activity against caspase-8 and -3 with poor selectivity for cathepsins [138]. Therefore, for effective clinical translation of caspase-based inhibitors in cancer, the incorporation of drug design linked with a detailed understanding of caspase function is more likely to become increasingly valuable for the identification of a new class of therapeutics.

## Conclusion

Numerous preclinical studies (in vitro and in vivo) over the years have suggested the role of caspases primarily as inflammatory and apoptotic mediators in the various pathologies. As a result, several caspase inhibitors have been patented targeting caspase inflammatory and apoptotic functions. However, their application is limited to preclinical studies due to the numerous challenges previously mentioned. While some studies have proposed novel therapeutic approaches using nanoparticle delivery systems and CRISPR/Cas9 gene editing to improve drug delivery and reduce drug-induced toxicity, and target individual caspases, respectively, these are still short-term solutions [139, 140]. This is because the potential of caspase inhibitory agents is further complexed by the crosstalk between alternate cell death and inflammatory pathways in the absence of caspase activity, which raises concerns on the long-term efficacy of caspase inhibitors and whether switching to alternate pathways heightens the risk of increasing cell death and inflammatory responses that may exacerbate the disease and needs to be more clearly established in preclinical models. Recent advances in the non-apoptotic and non-inflammatory functions of caspases suggest that caspase inhibition may alter more functions than intended. For example, caspase-8 has been shown to be an important regulator in maintaining a balance between apoptosis and necroptosis and is required for the suppression of necroptotic cell death [141, 142]. Evidence also suggests a role for caspase-8 in metabolism and immune response by the activation of the NF-κB pathway and the involvement in immune cell proliferation and differentiation [143, 144]. Likewise, caspase-2 and -3 were shown to influence metabolism independent of cell death with contradictory results suggesting the cleavage of the transcription factor, sterol-regulatory binding proteins (SREBPs), involved in cholesterol and fatty acid synthesis [145–148]. Caspase-1 has also been linked with metabolism by cleavage of SREBPs and the nuclear receptor, peroxisome proliferator-activated gamma, involved in insulin sensitivity and glucose metabolism [149–151]. However, these functions are not yet completely understood which poses a greater challenge for the development of effective inhibitors. Furthermore, in the absence of more detailed studies, the significance of caspases in apoptosis and inflammation, and non-apoptotic and non-inflammatory responses, or both functions in disease remains unclear. Nevertheless, the accumulated preclinical studies provide a lead for further investigations in understanding the exact roles of caspases (apoptotic/non-apoptotic and inflammatory/non-inflammatory), and alternate caspase-independent responses in disease states which importantly, will provide a better approach for targeting caspases and therapeutic advantage.

## Data Availability

The authors have no data to deposit on a repository.
